# A garter snake transcriptome: pyrosequencing, *de novo *assembly, and sex-specific differences

**DOI:** 10.1186/1471-2164-11-694

**Published:** 2010-12-07

**Authors:** Tonia S Schwartz, Hongseok Tae, Youngik Yang, Keithanne Mockaitis, John L Van Hemert, Stephen R Proulx, Jeong-Hyeon Choi, Anne M Bronikowski

**Affiliations:** 1 Ecology, Evolution and Organismal Biology Department, Iowa State University, Ames, IA 50011, USA; 2 BCBlab, Iowa State University, Ames, IA 50011, USA; 3 The Center for Genomics and Bioinformatics, Indiana University, Bloomington, IN 47405, USA; 4 School of Informatics and Computing, Indiana University, Bloomington, IN 47408, USA; 5 Ecology, Evolution and Marine Biology Department, University of California, Santa Barbara, Santa Barbara, CA 93106-9620, USA

## Abstract

**Background:**

The reptiles, characterized by both diversity and unique evolutionary adaptations, provide a comprehensive system for comparative studies of metabolism, physiology, and development. However, molecular resources for ectothermic reptiles are severely limited, hampering our ability to study the genetic basis for many evolutionarily important traits such as metabolic plasticity, extreme longevity, limblessness, venom, and freeze tolerance. Here we use massively parallel sequencing (454 GS-FLX Titanium) to generate a transcriptome of the western terrestrial garter snake (*Thamnophis elegans*) with two goals in mind. First, we develop a molecular resource for an ectothermic reptile; and second, we use these sex-specific transcriptomes to identify differences in the presence of expressed transcripts and potential genes of evolutionary interest.

**Results:**

Using sex-specific pools of RNA (one pool for females, one pool for males) representing 7 tissue types and 35 diverse individuals, we produced 1.24 million sequence reads, which averaged 366 bp in length after cleaning. Assembly of the cleaned reads from both sexes with NEWBLER and MIRA resulted in 96,379 contigs containing 87% of the cleaned reads. Over 34% of these contigs and 13% of the singletons were annotated based on homology to previously identified proteins. From these homology assignments, additional clustering, and ORF predictions, we estimate that this transcriptome contains ~13,000 unique genes that were previously identified in other species and over 66,000 transcripts from unidentified protein-coding genes. Furthermore, we use a graph-clustering method to identify contigs linked by NEWBLER-split reads that represent divergent alleles, gene duplications, and alternatively spliced transcripts. Beyond gene identification, we identified 95,295 SNPs and 31,651 INDELs. From these sex-specific transcriptomes, we identified 190 genes that were only present in the mRNA sequenced from one of the sexes (84 female-specific, 106 male-specific), and many highly variable genes of evolutionary interest.

**Conclusions:**

This is the first large-scale, multi-organ transcriptome for an ectothermic reptile. This resource provides the most comprehensive set of EST sequences available for an individual ectothermic reptile species, increasing the number of snake ESTs 50-fold. We have identified genes that appear to be under evolutionary selection and those that are sex-specific. This resource will assist studies on gene expression and comparative genomics, and will facilitate the study of evolutionarily important traits at the molecular level.

## Background

Comparative studies are invaluable for understanding the evolution of complex traits. The reptile lineage has given rise to both a metabolically endothermic group (birds) and diverse ectothermic groups [1], thus providing a comprehensive system for comparative studies of metabolism, physiology, development and aging [2]. Ectothermic reptiles (e.g. turtles, crocodilians, tuatara, lizards, and snakes) exhibit extreme plasticity in their ability to modulate their metabolism in response to external stresses such as thermal and food stress [2-5]. Furthermore, they show extraordinary diversity in body structure (e.g. turtle shells, squamate limblessness), sex determining systems (e.g. temperature-dependent vs. genotypic), and sexual dimorphism. Snakes, in particular, have evolved dramatic evolutionary adaptations (e.g. venom, limblessness) and have been useful models for evolution and ecology [6], but the pursuit to understand these evolutionarily important traits at the molecular level has been limited by the molecular resources available. The heightened interest to utilize reptiles in molecular genetic studies is signified by the first two on-going ectothermic reptile genome projects (Anolis lizard and painted turtle) conducted by NIH [7,8], and the additional five ectothermic reptile genomes selected to be sequenced by BGI [9]. As well, the recent publications of viper venom gland transcriptomes [10], and the first ectothermic reptile linkage map (crocodile) [11] emphasize that developing genomic resources for this interesting group is of utmost importance. First glimpses into the evolution of reptile genomes (endothermic and ectothermic) have revealed unique genomic attributes such as microchromosomes, the evolution of gene structure and gene synteny [12-14], as well as dramatic evolutionary changes in functionally important genes [15].

Ultimately we are interested in understanding how reptiles use their genomes in sex-specific ways to respond to environmental and evolutionary pressures, and how these responses affect reproduction and aging. A necessary first step is to develop and characterize molecular resources for a species of interest. The main focus of this paper is to develop a garter snake transcriptome so it can be used as a reference for future studies. The western terrestrial garter snake (*Thamnophis elegans*) is an emerging model for understanding the molecular basis for the evolution of life-history trade-offs between cellular maintenance and longevity versus growth and reproduction [16]. Closely related populations of this species, found in the Sierra Nevada Mountains, harbor two ecotypes that contrast in physical appearance, physiological and behavioural stress response, natural lifespan, and reproductive traits. These two ecotypes can be contrasted as either "fast-living" or "slow-living" based on the overall life-history [17-20]. These ecotypes derive from both genetic and environmental differences [17], and selection acts strongly on divergent traits despite low levels of gene flow among these populations [21,22]. Additionally, males and females have different costs associated with reproduction, which is expected to cause sexual conflict at the genomic level. This conflict may be resolved through differential regulation of how these sexually antagonistic genes are utilized in each sex [23].

The paucity of molecular resources available for the garter snake and other ectothermic reptile species has limited our ability to identify the genetic basis for evolutionarily important traits, thus providing the inspiration behind this project. The application of new sequencing technologies, such as pyrosequencing, to traditional ecological models expands the horizon for ecological genomic studies [24-29]. Indeed, we are on the verge of elucidating the genetic basis of ecologically and evolutionarily relevant traits in natural populations. Here we apply this technology to develop a large scale, multi-tissue, multi-individual transcriptome using massively parallel sequencing with two goals in mind. Our first goal is to develop a molecular resource for the garter snake and make it available to the scientific community. This resource is a generalized transcriptome (i.e., RNA was pooled across ecotypes, populations, individuals, and tissues) for use as a reference for future studies. Our second goal is to identify sex-specific differences in the presence/absence of expressed transcripts by identifying transcripts that were present in the normalized library of one of the sexes but not the other. These data are accessible through the Garter Snake Transcriptome Browser at Indiana University CGB https://lims.cgb.indiana.edu/cgi-bin/gbrowse/telegans_bronikowski_2/, the Bronikowski Lab Data Server http://eco.bcb.iastate.edu/, and through NCBI Short Read Archive (SRA010134).

## Results and Discussion

### Sampling and 454 GS-FLX Titanium Sequencing

Our goal in sampling was to maximize the identification of unique transcripts, while capturing the diversity of expressed transcripts across tissues, individuals, populations, and stress conditions. Therefore, keeping male and female samples separate, we pooled RNA from 35 garter snakes (*T. elegans*) of varying sizes/ages (at least 1 year old) into two sex-specific RNA samples (sampling details in Additional file 1). The snakes were both laboratory-born and field-caught from seven focal populations of the Sierra Nevada Mountains in California. These sex-specific pools of RNA were used to develop normalized cDNA libraries that were sequenced on separate halves of a GS-FLX Titanium (Roche/454 Life Sciences) PicoTitre plate. We also obtained an extra quarter plate of male library reads for quality control assessment. This resulted in 446 Mbp of sequence data, 1.24 million reads (i.e. expressed sequence tags) averaging 366 bp in length after cleaning (Table 1; see Additional file 2 for size distribution of reads). The cleaned reads have been deposited in the NCBI Short Read Archive (SRA010134).

**Table 1:** Summary statistics for 454 sequencing and de novo assembly.

	Female	Male	Total
Total number of raw reads	538,706	753,163	1,291,869

Number of reads after cleaning	507,466	730,814	1,238,280

Amount of cleaned data	207 Mbp	246 Mbp	453 Mbp

Average length of cleaned reads (bp)	408	337	366

NEWBLER contigs (large > 500 bp)	NA	NA	82,134 (30,520)

NEWBLER contig bp total	NA	NA	48 Mbp

NEWBLER contig length (bp)	NA	NA	90-10,680 (avg 581)

Average coverage (NEWBLER)	NA	NA	7.6×

Reads placed (NEWBLER)	NA	NA	1,035,854

Singletons (after NEWBLER)	NA	NA	134,971

MIRA contigs	NA	NA	14,245

Reads placed (MIRA)	NA	NA	33,093

Singletons mapped by BLAST to contigs	NA	NA	5846

Reads discarded^1^	NA	NA	70,926 (5.7%)

Remaining Singletons	NA	NA	92,561 (7.5%)

Total reads placed	NA	AN	1,074,793 (86.8%)

Total number of contigs	NA	NA	96,379

SNPs	NA	NA	95,295

INDELs	NA	NA	31,651

454 GS-FLX Titanium chemistries were used to sequence normalized sex-specific pools of cDNA.

^1^Reads were discarded either due to redundancy (3461) or for quality control by NEWBLER or MIRA during the assembly process (67,455 and 10 respectively).

### Assembly and Annotation

**Figure 1:**
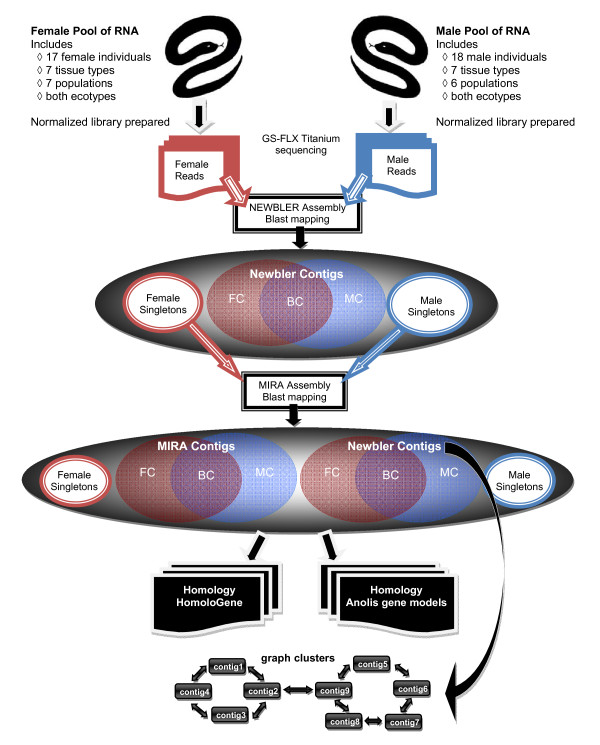
**Flow diagram of the assembly procedure**. Female and male normalized pools of cDNA were sequenced separately using Roche 454 GS-FLX Titanium chemistries. NEWBLER was initially used to assemble the cleaned reads into contigs that could be classified into three categories based on the sex-of-origin of the reads: Female Contigs (FC, contigs made only from female reads); Male Contigs (MC, made only from male reads); Both Contigs (BC, contigs made from both male and female reads). The remaining male and female singletons went through additional assembly using MIRA. After each assembly process an attempt was made to map the remaining singletons to the contigs using Blast. The contigs and singletons were clustered based on homology in the HomoloGene database or to the Ensemble annotated gene models from the Anolis lizard draft genome (AnoCar1.0). The NEWBLER contigs were put into graph-clusters based how reads were split and assigned to different contigs during the NEWBLER assembly process (see Additional file 3 for full description). In the graph-clusters the contigs (nodes) are linked by reads (edges) that were split between the contigs.

The male and female reads were pooled for assembly, but the sex-of-origin for each read was tracked, which allowed contigs to be categorized as containing reads from both sexes, from males only, or from females only (Figure 1). Relative to the garter snake, the sequenced (draft) genome closest in evolutionary relationship is the Anolis lizard (*Anolis carolinensis*), which shared its most recent common ancestor with snakes ~215 million years ago [30]. The next evolutionarily closest species with a sequenced genome is the chicken (*Gallus gallus*), which shared its most recent common ancestor with snakes ~285 million years ago [31]. We used 454 gsMapper 2.3 to map the garter snake reads to the draft Anolis lizard and the chicken genomes. Of cleaned reads, 773,997 (62%) and 536,900 (43%), respectively, were mapped, even with the minimum percent identity of 80%. Those mapped reads were assembled to 255,211 and 175,279 contigs for the lizard and chicken genomes, respectively.

For *de novo *assembly, reads were assembled using GS *de novo *Assembler (NEWBLER v2.0.00.22; Roche) resulting in 82,134 contigs - with 84% of the reads being placed - with an average depth of 7.6× coverage (Table 1). We then used the program MIRA [32] for additional assembly of the remaining singletons. This resulted in 33,338 singletons (25%) being assembled into an additional 14,245 contigs. The remaining singletons were mapped to contigs in order to remove sequence redundancy. Overall, 86.8% of reads were assembled into 96,379 contigs with 7.5% of the reads remaining as singletons, and 5.7% of reads being discarded (Table 1). Because many more of the reads were place with the *de novo *assembly, we use the *de novo *assembly for all subsequent analyses.

The percentage of reads assembled *de novo *is similar to other studies that have applied pyrosequencing to non-model organisms [25,27,33]. The large number of contigs is likely due to the extensive diversity in the initial RNA samples - pooled across individuals and populations - in the form of sequence variants and alternative splicing. Different organs, different sexes, and different environmental/stress conditions are known to produce extensive alternative spliced transcripts in vertebrates [34]. This variation causes misalignments between reads arising from the same genomic region, preventing correct assembly by most algorithms. For this reason, NEWBLER was used for assembly because it splits reads at the boundaries of variation in order to build contigs reflecting both static and variable regions [35] (see Additional file 3 for graphical representation of Newbler-split reads and assembly).

**Figure 2:**
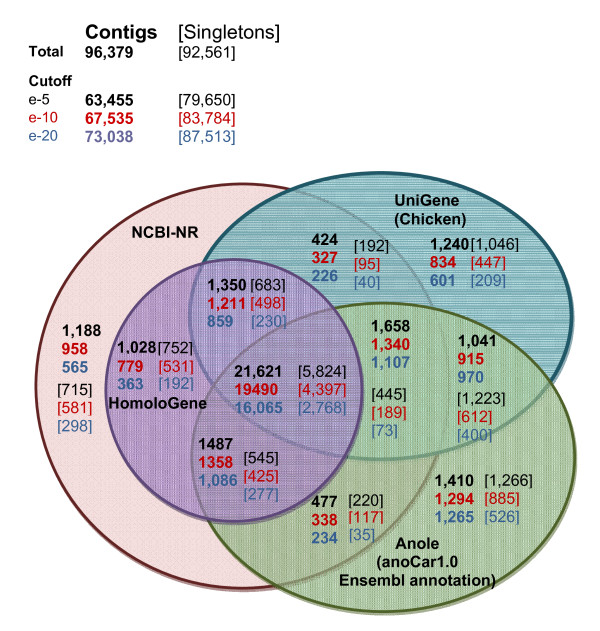
**Venn diagram of BlastX results**. The number of contigs (both NEWBLER and MIRA) and singletons that found a homologue when Blasted against UniGene (chicken), HomoloGene, NCBI-NR databases and the draft lizard (*Anolis carolinensis*) genome and transcriptome (anoCar1.0) at three different e-value cut offs.

The NEWBLER contigs, the MIRA contigs, and the singletons were compared to three reference databases for annotation using BlastX: NCBI non-redundant protein database (NR); NCBI HomoloGene; and UniGene (Chicken). The sequences were also compared to the 18,031 Ensemble annotated genes (17,672 coding and 359 pseudogenes) from the Anolis lizard draft genome (AnoCar1.0; http://uswest.ensembl.org/Anolis_carolinensis/) using tBLASTx. Over 24% of all sequences (contigs and singletons) identified a homologue in at least one of these reference databases at e-value 1e^-5 ^(Figure 2: annotated data can be downloaded from http://eco.bcb.iastate.edu/). As expected with longer sequences, a higher proportion of the contigs found an identity (34%) relative to the singletons (13%). Most of the contigs that found an identity in one database were identified in all four of the reference databases (Figure 2). This subset of contigs likely contains the more conserved genes that are well characterized and thus found in all of these databases. The number of contigs matching a homologue is similar or higher to other sequencing studies on non-model organisms [28,33].

The number of unique homologous genes identified in each database ranged from 26,232 in NCBI-NR to 12,953 in UniGene (Chicken) (Table 2). The BlastX results against the HomoloGene database and the Anolis lizard Ensemble annotation likely give the most realistic estimate of the number of unique genes in our dataset for which we could assign a homology ID, approximately 13,000. Naturally, the more quickly evolving genes and the snake-specific genes would be unlikely to find homologues in any of these databases. Thus, undoubtedly, there are uncharacterized genes yet to be discovered in the other 76% of the sequences for which we could not assign an ID based on these reference databases. ORF predictions indicate that an additional 97% of the non-annotated sequences had a predicted open reading frame of at least 30 bp, which suggests that these were transcribed from protein-coding genes. The majority of the GO annotations assigned to the snake sequences were for the biological processes of metabolism and regulation, although there were also a smaller number of sequences assigned to reproduction, behaviour, and stress response (see Additional file 4 for GO pie graphs).

**Table 2:** The number of unique homologues in each database identified through BlastX using four e-value cut-offs. Databases as of January 2010.

	1e^-5^	1e^-10^	1e^-20^	1e^-50^
NCIB-NR	26,232	23,710	19,485	12,197

UniGene (Chicken)	12,953	12,317	11,348	9012

*Anolis *Ensembl annotated genes	13,923	13,533	12,806	10,515

HomoloGene	13,346	12,876	11,803	8771

Using tBlastX, 55,715 snake transcripts (contigs and singletons) were also mapped to the lizard draft genome (AnoCar1.0). To identify 5' and 3' UTRs and non-coding RNAs, these matches to the Anolis genome were compared to the matches to the AnoCar1.0 Ensemble annotation of coding (18,031) and non-coding (2939) RNA. We identified 2322 snake transcripts that contained 5'UTR (286 matched to 5'UTR only, the rest matched 5'UTR along with protein coding sequence and/or intergenic sequence), and 3680 that contained 3'UTR (1018 matched to 3'UTR only, the rest matched 3'UTR along with protein coding sequence and/or intergenic sequence). Furthermore, 2,534 of our snake transcripts matched the Anolis non-coding RNAs, and 36,188 matched other intergenic regions of the Anolis genome and therefore may also be non-coding RNAs (see Additional file 5 to access these transcripts). A Snake Transcriptome Browser (GBrowse) of the snake transcripts mapped against the Anolis draft genome (AnoCar1.0) has been set up to visualize the data, and to access the assembled and annotated data files https://lims.cgb.indiana.edu/cgi-bin/gbrowse/telegans_bronikowski_2/.

To check for additional non-coding RNAs, we used RNAmmer 1.2 [36] and tRNAscan-SE 1.23 [37]. One contig was predicted to be 8 S rRNA, although this was not supported by the homology search through NCBI non-redundant protein sequences. Thus, it is likely a false prediction. Five singletons were identified as tRNAs: two sequences for tRNA**^Asp^**, one for tRNA**^His^**, one for tRNA**^Lys^**, and one sequence for tRNA**^Ser^**. Additionally, pseudogenes were predicted from 5 contigs and 11 singletons.

### Phylogenetic assessment of BLAST results

**Figure 3:**
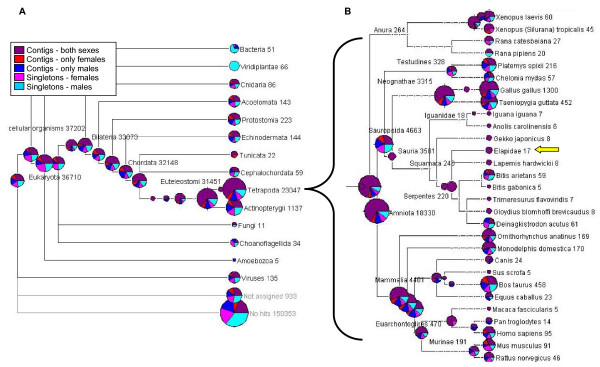
**Taxonomic assignments of sequences**. Assignment of each class of sequences to the least common ancestor of their BlastX hits (e-value = 1e^-5^) using MEGAN. A) Distribution of assignments on the NCBI taxonomy tree. The pie graphs are proportional to the number of sequences assigned to that node, whereas the numbers are the accumulative sum of sequences assigned within that subclade. B) Expansion of the Tetropoda node. The yellow arrow indicates the placement of the garter snake on the taxonomic tree.

We use the phylogenetic distribution of the Blast hits to identify off-target sequences and as a qualitative assessment of the assembly. Because we indiscriminately used all RNA isolated from six tissue types and blood, which would contain disease and commensal organisms, we expected our dataset to contain off-target species sequences that did not originate from the garter snake genome. To identify these off-target sequences we used the metagenomics program MEGAN [38]. MEGAN assigns a sequence to the lowest common ancestor of all its Blast assignments at a particular cut-off e-value. Thus sequences placed at the deeper nodes of the phylogeny represent more conserved genes relative to the genes on the leaves the tree that are more specific to that species. We used the output from BlastX (e-value = 1e^-5^) against the NCBI-NR for the contigs and singletons (categorized based on sex-of-origin) to map the hits on the NCBI taxonomic tree (Figure 3). We identified 979 sequences (0.5% of the dataset) that were likely from off-target species including 51 that assigned to bacteria, 135 that assigned to viruses, and 11 that assigned to fungi. Interestingly, there was a bias towards bacterial sequences originating from the male reads suggesting the possibility that one or more of the males in our sample may have had a high bacterial infection load.

Equal proportions of sex-specific contigs and singletons were assigned to the nodes of the taxonomic tree, which implies that our ability to map to NCBI was not biased towards one or the other sex (Figure 3). Overall, 88% of the assigned sequences were assigned to chordates or a lineage within chordates; and 62% were assigned to tetrapods or a lineage within tetrapods (Figure 3). The placement of sequences at a particular node is highly dependent upon the relative abundance (or presence) of sequences available in NCBI-NR from each taxonomic group. Although there are far fewer protein sequences from reptiles (*Sauropsida*) in NCBI-NR compared to mammals (*Mammalia*), both lineages had roughly equal number of genes assigned within them. The species with the most number of genes matched was *Gallus gallus *(chicken), which is the evolutionarily closest species with a completed genome in the NCBI-NR protein database.

As an additional evaluation of the quality of our assembly and sequencing, we identified potential chimeric sequences that are made-up of two unique sequences that have been concatenated such that the sequence had two highly significant (< 1e^-20^) NCBI-NR hits to different genes, which aligned to different ends of the contig or singleton. We identified 24 contigs and 2 singletons that had such signatures (i.e., representing < 0.025%). Of these, two of the contigs were adjacent mitochondrial genes representing a correct assembly as the mitochondrial genome is known to be transcribed in large multi-gene segments [39]. The other 22 contigs and 2 singletons seemed to be true chimeras based on visual inspection of the BlastX hits. These chimeric sequences could be due to misassembly, chimerization during the library construction, true biological gene fusion events, or errors in GenBank. Overall, the phylogenetic distribution of the BlastX hits and the low percentage of chimera sequences provide qualitative support for our assembly.

### Additional clustering

Complex vertebrate transcriptomes are characterized by numerous alternatively spliced transcripts and transcripts from duplicated genes [34,40] making *de novo *assembly difficult. We used two methods for additional clustering of the contigs and singletons into reference gene sets: clustering based on homology, and clustering based on contig-graphs (Figure 1). Homology clustering uses BlastX against reference databases to group contigs and singletons into clusters that are likely to have originated from the same gene. Homology clustering of contigs and singletons based on the HomoloGene database produced 8771 - 13,346 homology clusters, depending on the e-value cut-off used (Table 2). At e-value 1e^-20^, each of these homology clusters contained 1 to 93 contigs with an average of two contigs (see Additional file 6 Panels A and B for distributions of contigs and clusters). Additionally, ~2000 of the contigs assigned to two or more homology clusters (HomoloGene accession); these contigs likely belong to gene families or are contigs with a strong domain found in multiple HomoloGenes. For example, contig00258 assigned to four HomoloGene clusters, each of which contains an ubiquitin conjugating enzyme E2 catalytic domain (UBCc). Homology comparisons to the draft gene models from the lizard genome had a smaller range of minimal gene sets (Table 2). These homology clusters contain contigs and singletons that represent alternative alleles, alternatively spliced transcripts, and non-overlapping contigs from the same gene and possibly closely related genes in the same family (particularly at the lower e-value).

**Figure 4:**
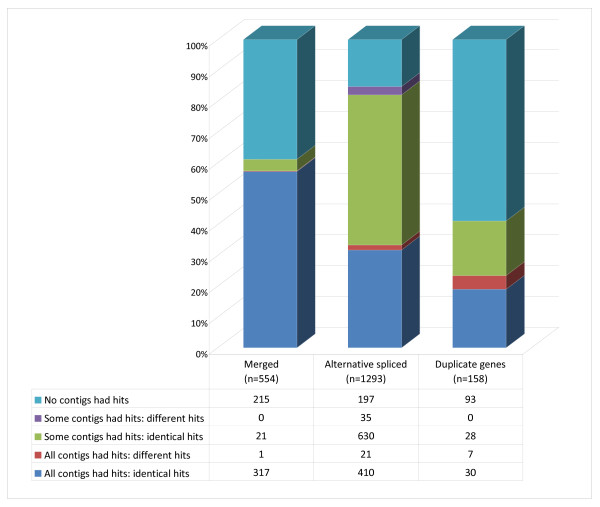
**BlastX results for the components identified in the graph-clusters**. Classification of the results of the BlastX NCBI-NR hits for each contig in each graph-cluster component, presented as a proportion of each type of component. "Merged" are components have nodes (i.e. contigs) that were merged due to sequence similarity. "Duplicate genes" are components that consist of pairs of contigs from duplicated genes. "Alternative spliced" are components that consist of nodes that are predicted to contain alternatively spliced exons from the same gene (after merging contigs and collapsing duplicated contig pairs).

Output clusters from the contig-graphing approach were used to group NEWBLER-generated contigs based on read-assembly information (see methods and Additional file 3 for a complete description). For alternatively spliced transcripts, highly diverged alleles and repeat regions of duplicated genes, NEWBLER (454 GS Assembler) splits reads into separate contigs. During assembly, split reads are tracked and that information can be used to reconstruct how contigs may be related. This can be illustrated graphically using networks (i.e. graph-clusters) such that nodes in the graphs represent contigs and an edge between two nodes represents a read split between two contigs. This clustering is independent of the contigs' homology to other databases, and allows for further identification of divergent alleles, alternatively spliced transcripts, and gene families based on the structure of the network and nucleotide similarity of the contigs in the network (see Additional file 3 for more details of this method). We constructed 6,860 graph contig clusters containing 27,860 NEWBLER contigs. The graph-clusters contained on average four contigs (range 2-95) (see Additional file 6 panel C for distribution of contigs in graph-clusters). This clustering method can be used to further merge variable alleles of the same gene, construct alternatively spliced transcripts, and identify gene families. Within these graph-clusters, we identified 554 components that are predicted to represent highly divergent alleles of the same gene that can then be merged, 1,293 components that are predicted to represent alternative splicing events, and 158 components (pairs of duplicated contigs) that are predicted to represent duplicated genes (Figure 4).

Evaluating the BlastX NCBI-NR results of the contigs within each graph-cluster component revealed differences among the classes of components. As you would expect from alleles of the same gene, all the contigs from merged components (highly variable alleles of the same gene) were more likely to match the same gene in NCBI-NR than were contigs from alternatively spliced or duplicated gene components. Likewise, contigs in the 158 duplicated gene components were more likely to match different genes in NCBI-NR than either the merged components or the alternative spliced components (Figure 4).

**Figure 5:**
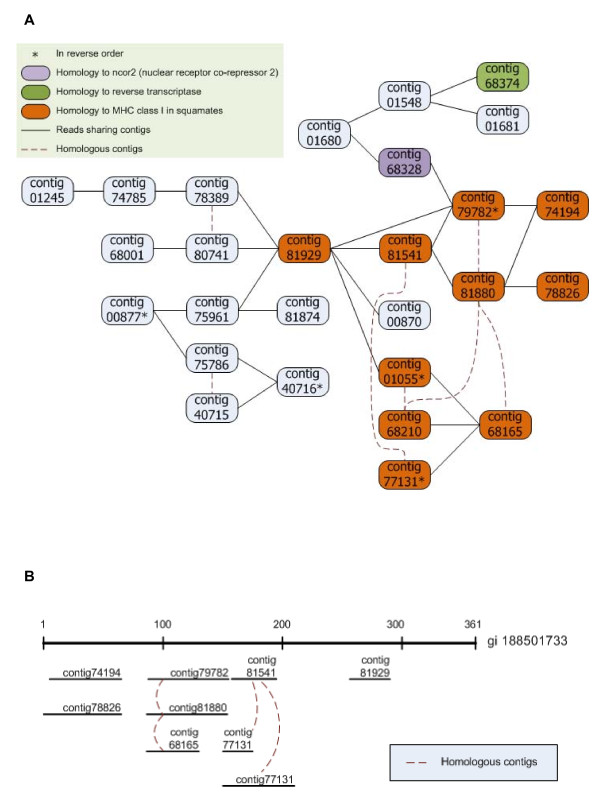
**MHC class I graph-cluster**. The MHC class I graph-cluster (cluster 05625) that contains allelic variation, alternative spliced transcripts and potential gene duplication(s). A) The nodes of the network cluster represent contigs, solid lines (edges) represent reads split between the two contigs during assembly by NEWBLER, and the dotted lines represent homologous contigs representing either divergent alleles from either the same or duplication genes. The orange contigs were identified as homologues of MHC class I in squamates (lizards and snakes), the light blue contigs had no hits in NCBI-NR. B) Alignment of the contigs to their BlastX hit in the NCBI-NR database, MHC class I antigen from Iguanid lizard, *Conolophus subcristatus*. Again, dotted lines represent homologous contigs representing either divergent alleles from either the same or duplication genes.

As an example of the usefulness of this graph-clustering method, we highlight graph-cluster05625 (Figure 5) that consists of 27 contigs, of which nine contigs have identical hits in NCIB-NR, three contigs have different hits in NCBI-NR, and 15 contigs have no hits in NCBI-NR. Ten of these NCBI-NR matches are to the hypervariable immunological gene MHC class I, which is well documented as being under diversifying selection in other species [41]. This clustering method allowed us to identify additional contigs (not identified by our homology searches) that represent multiple alleles, alternatively spliced transcripts and a potential gene duplication event for a highly important immunological gene complex.

### Sequence variants

**Figure 6:**
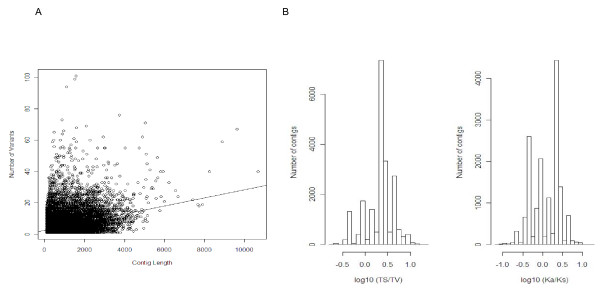
**Variants**. A) Regression of number of the variants on contig length for each contig with at least one variable site. B) Histograms of the log10 distribution of the TS/TV ratios for contigs containing SNPs, and the log10 distribution of Ka/Ks ratios for the contigs containing SNPs in predicted ORFs. Contigs with TS/TV < 1 (0 on log10 scale) and Ka/Ks > 1 (0 on log10 scale) are suggested to be under diversifying selection in these populations of garter snakes.

One of our goals for including diverse individuals from multiple populations was to identify sequence variants (SNPs and INDELS) that would be useful in future studies. At a Bayesian probability of 90% we identified 126,946 variants: 95,295 SNPs and 31,651 INDELs in either single contigs (23,615) or across contigs that had been merged into graph-clusters (549). Over 110,000 of these variants had >99% probability of being a true variant rather than a sequencing error. Of the single contigs that had variants, the average number of variants per contig is 5.16 (SNPs = 3.84, INDELs = 1.32); with an average rate of one variant per 200 bp window (see Additional file 7 for details on variants). The number of variants (SNPs and INDELs) per contig had a weak but significant correlation with contig length (F = 1980, DF = 23,615, R^2 ^= 0.07, p-value < 0.01: Figure 6 panel A), with the scatter suggesting that some contigs represented highly conserved genes with low variation and some contigs with high levels of variation for contig length.

The degree and type of variability within a contig can indicate selection acting on the gene. As expected, large contigs with few variants tended to be from highly conserved genes including, MACF1 (microtubule-actin cross-linking factor 1), and myosin. We identified 236 contigs that were highly variable (in the 99^th ^percentile for highest ratio of variants per contig length) and, as expected of quickly evolving genes, few of these (19%) had homology hits in the BlastX searches, but 98% of them had predicted open reading frames (ORF) larger than 30 bp. This size criterion suggests that the ORFs were within true protein coding sequences. Of the 45 contigs that were highly variable and had a BlastX hit in NCBI-NR, at least 8 were from off-target sequences and 11 were associated with transposable elements, but there were also many sequences of interest that are known to be hypervariable and/or quickly evolving in vertebrate taxa. These included three immunological genes (MHC class I, complement factor-H related protein, interferon regulatory factor 7), a snake venom gene (venom factor 1), and a predicted pheromone receptor (see Additional file 8 for full annotation data on these genes of interest). Interestingly, there were also two highly variable genes involved in lipid metabolism or lipid oxidation: peroxisomal long-chain acyl-CoA thioesterase, and fatty acid desaturase 1. These genes may be particularly relevant for future studies on the evolution of metabolism and stress response in these garter snake populations.

Mutations to a nucleotide of a similar structure (i.e., TS: transitions - mutations from a purine to a purine, or from a pyrimadine to a pyrimidine) occur more often then transversions (TV: mutation from a purine to a pyrimadine or vice-versa). Thus, a TS/TV ratio <1 may reveal sequences subjected to diversifying selection [42]. We found 73, 836 TSs and 21, 459 TVs in this dataset. We identified 2, 165 contigs with a TS/TV <1. For SNPs within predicted coding regions, we determined whether they were non-synonymous polymorphisms (Ka) that changed the amino acid, or were synonymous polymorphisms (Ks). Overall, 29, 883 of the SNPs found in a coding region were non-synonymous and 23, 252 were synonymous. We found 8, 417 contigs (8.7% of all contigs) with a Ka/Ks ratio >1. This indicates that mutation(s) have changed the amino acid sequence more than would be expected under a neutral model, and that these genes may be under diversifying selection within or among these snake populations. The distributions of TS/TV and Ka/Ks are in Figure 6A. Of most interest are the 16 contigs at the intersection of Ka/Ks >1, TS/TV <1, and the 99^th ^percentile of highly variable contigs. Only three of these could be assigned a putative identification based on homology: the immune complement factor-H related protein, fatty acid desaturase 1, and a KRAB transcription factor. Revisiting the MHC class I graph-cluster05625 (Figure 5) that consists of 27 contigs, of the 20 contigs had variants 10 had Ka/Ks > 1. As predicted above, this further supports diversifying selection across this complex. Additional highly variable genes with high Ka/Ks ratios are likely to be targets of diversifying selection, potentially diversifying across the populations (or ecotypes) of the garter snakes.

### Comparison between female and male

When the male and female reads were pooled and assembled into contigs, each read was tracked by the sex from which it was generated. Thus, the contigs and singletons could be classified on the origin of its reads. Focusing only on the sequences for which we could assign an ID based on homology, NCBI-NR BlastX hits (1e^-50^) were summarized based on whether they were unique to females (i.e., found only in female contigs and/or female singletons), were unique to males, or were composed of reads from both sexes. In this way we identified 190 genes (195 contigs) that were only present in the mRNA sequenced from one of the sexes (see Additional file 8 for full annotation data on these genes of interest). Of these 190 genes, 84 were expressed only in females and 106 were expressed only in males. While this is a relatively low number of genes that are sex-specific, recall that our sex-specific pools of RNA included seven tissue types and were normalized in order to maximize the number of unique transcripts. Therefore, these data are not quantitative differences in expression in a particular tissue type, but are presence/absence data across all seven tissues. Most other studies that look at sex-specific differences use microarrays (or RNA-seq) on non-normalized libraries from a particular tissue and thus have quantitative data to show difference in the levels of expression between the sexes. Indeed it is at the quantitative level that most genes are biased in their expression between the sexes [43]. Additionally, most genes that are biased in their expression between sexes are specific to a particular tissue. For example, a microarray study on chicken brain, gonad, and heart, identified ~13, 000 genes that had quantitative differences in one particular tissue, but only four genes were significantly different in all three tissues [44]. As well, sex-specific genes are typically quickly evolving; thus, homologues may not be present in the available databases [43].

The female-specific sequences were enriched for GO terms for "biosynthetic processes" relative to the male-specific sequences (FDR < 0.006, p-value < 0.0002: see Additional file 9 for distribution of GO terms by sex). Although many of these sex-specific genes were uncharacterized or classified as "predicted coding genes", some met our expectations for being sex-specific. For example, two of the male-specific genes are known to be involved in spermatogenesis (SPATA18, SPATA22) and two in sperm motility (CATSPERG, CATSPER2). Additionally, two female-specific genes are known to be involved in hormonal signalling and regulation (GnRH receptor and Irg1) [45-47]. Five of these 190 sex-specific genes also had a TS/TV ratio ≤ 1, male-specific: BTBD16, CATSPERG, CATSPER2; and female-specific: RAG1 and CDX4. Additionally, 13 of the sex-specific genes had a Ka/Ks ratio > 1, three of these overlapped with those that had a low TS/TV: male-specific CATSPERG, female specific CDX4 (also on the human X chromosome), and female specific RAG1.

These analyses suggest the CATSPER genes maybe under strong selective pressure. The CATSPER genes (1-4) produce proteins that form an ion channel that is specific to the sperm flagellum, and are required for hyperactivated motility during the fertilization process [48-50]. Mutations in the CATSPER genes cause infertility in mice and humans [49-51]. The CATSPERG gene encodes an additional protein that is associated with the CATSPER ion channel [52]. As with many reptiles, garter snakes mate multiply and have multiple paternity litters [53], which could lead to sperm competition and selective pressure on sperm function and motility. Further studies are needed to test the role of diversity in the CATSPER genes in reproductive fitness and sperm competition in this snake system. Although we hypothesize that these male-specific genes associated with sperm production and fertilization are being expressed in the gonads, we cannot address the question of tissue-of-origin with these data at this time. However, we are currently looking at gene-expression differences in individual-based and tissue-specific libraries.

Both snakes and birds have ZW/ZZ sex chromosomes, in which the female is the heterogametic sex. The chicken and snake are separated by ~285 million years and it has been well documented that reptile sex chromosomes have undergone drastic rearrangements over that time [54]. Therefore, whether any snake sequences aligned to the chicken sex chromosomes would be a very interesting result. Indeed, the snake sequences aligned to 15 genes on the chicken Z chromosome. Interestingly, four of these had female-specific expression and two had male-specific expression (see Additional file 8 for full annotation data of these genes of interest). It remains to be determined whether these genes reside on the garter snake Z (or W) chromosome. Equally interesting is that one gene known to be on the human X chromosome, CDX4, had female specific expression, had a TS/TV < 1, and a Ka/Ks > 1 suggesting it may be a sex-conflict gene that is quickly evolving in these snake populations.

Because the cDNA libraries used for sequencing were normalized, the identification of sex-specifically expressed genes is based on presence/absence rather than quantitative measures. Some of the genes identified here as sex-specific genes are likely under sex-biased expression, potentially the result of sex conflict resolved at the level of gene expression. Additionally, some of these sex-specific genes may reside on the garter snake sex chromosomes. Additional large-scale studies at the quantitative level will verify the sex-specific expression of these genes.

## Conclusions

We have successfully sequenced the first large-scale, multi-organ transcriptome for an ectothermic vertebrate using pyrosequencing and *de novo *assembly. In the process, we use a method for graphically clustering contigs after NEWBLER assembly that allowed us to identify divergent alleles, alternatively spliced transcripts and gene families. We have identified a number of interesting genes that are sex-specifically expressed and/or that are predicted to be quickly evolving that beg for additional investigation. These are the starting points for genetic studies on evolution of metabolic and immune function, sexual conflict resolution, as well as the evolution of sex chromosomes.

This transcriptome is the most comprehensive set of published EST sequences available for an individual ectothermic reptile species. It has increased the number of nucleotide ESTs available for ectothermic reptiles 5×, and for snakes 50×. Additionally, we have identified over 100, 000 high confidence variants (SNPs and INDELs) that can be used for population genetic studies, and quantitative trait mapping in this and related species.

These sequence data are a tool for future gene expression experiments, and comparative transcriptomic, genomic, and metabolomic studies. They can assist interested researchers to address evolutionary- and ecological-genomic questions in this and other reptile species. Ongoing and future studies can use this generalized transcriptome as a reference for mapping quantitative expression and sequence data from experiments that use, for example, short-read sequencing technologies. By combining these new sequencing technologies our labs hope to gain insight into how these snake life-history ecotypes have evolved, as well as how sex-conflict genes evolve. Overall, this is a valuable resource for the study of evolutionary important traits at the molecular level.

## Methods

### Sampling

A total of 35 western terrestrial garter snake (*Thamnophis elegans*) individuals of varying sizes/ages (at least 1 year old) were included in this transcriptome (17 females and 18 males: see Additional file 1 for details on sampling). The snakes were either laboratory-born and raised or field-caught from seven populations at the northern end of the Sierra Nevada Mountains in Lassen County, California, which included the two life-history ecotypes as has been previously described [2,18,55]. Adult field-caught snakes were shipped live to Iowa State University. The laboratory snakes were 2-year old animals that had been used in a thermal experiment. Within 10 minutes of euthanizing, all organs and blood were either snap-frozen in liquid nitrogen and stored at -80°C, or put in RNA-later and kept at room temperature for 24 hours and then stored at -20°C. Organs included brain, gonads (from sexually mature individuals), heart, kidney, liver, spleen and blood (ISU IACUC 3-2-5125-J).

### RNA isolation

Individual snake samples were pooled by tissue type and by sex for total RNA isolation using TriReagent and cleaned-up using Qiagen RNAeasy columns. The TriReagent manufacture's protocol was followed, but instead of precipitating the RNA, the supernatant from the chloroform step was added (0.75:1) to the RLT/BME buffer from the Qiagen RNAeasy kit. From this point on the Qiagen protocol was followed. Quality of the RNA was verified with a Bioanalyzer nanochip (Agilent). Total RNA quantity was determined by both the Bioanalyzer nanochip and a Nanodrop. RNA from each tissue type was pooled in equal amounts into their respective Male and Female samples, and concentrations determined by fluorometry using Quant-iT OliGreen (Invitrogen).

### Library preparation and sequencing

Library preparations for GS FLX Titanium (Roche/454 Life Sciences) sequencing were developed in the Center for Genomics and Bioinformatics, Indiana University based partially on methods for use in GS FLX standard sequencing described in Meyer *et al*. [25], with modifications (K. Mockaitis, unpublished). Briefly, cDNA was synthesized from 630 ng of each total RNA pool (male, female) in a manner similar to Clontech™SMART protocols, using primers optimized for the 454 sequencing process, and amplified by PCR to generate dsDNA. For partial normalization to reduce sequence coverage of high copy number transcripts, amplified cDNA was subjected to controlled in-solution hybridization and double-stranded nuclease (DSN) digestion using the Trimmer Direct kit (Evrogen) after reaction optimization. Normalized DNA was then fragmented by sonication, and ends enzymatically blunted and ligated to customized 454 adaptors. Amplification of ligation products exploited adaptor-mediated PCR suppression [25]. This method induces homo-adapted fragment hairpins, thereby severely limiting amplification of mis-ligated products. All amplification steps utilized high-fidelity polymerases. Final libraries were size selected by excision of the 500-800 bp fraction from agarose gels.

Emulsion PCR reactions were performed according to the manufacturer (Roche/454 Life Sciences). To optimize pyrosequencing throughput, prior to sequencing final libraries were titrated by emulsion PCR bead enrichment. Sequencing of male and female libraries was performed in parallel on a picotitre plate according to the manufacturer, and yielded 259 Mb (male) and 219 Mb (female) of sequence data in 1, 291869 reads with an average of 386 nts in length.

Sequencing adapters (A and B) were automatically removed from the reads using the signal processing software (Roche/454 Life Sciences). The reads were further cleaned and the adaptors removed by a program developed in-house at CGB, Indiana University http://sourceforge.net/projects/estclean/. After cleaning, sequences ≤30 bp were removed from the dataset. Thus the final cleaned dataset before assembly contained 1, 238, 280 reads with an average length of 366 nts (Table 1).

### Assembly and annotation

The pooled reads were mapped to the lizard and chicken genomes using the GS mapper v2.3 with 80% identity. For *de novo *assembly, the reads were assembled into contigs using the GS *de novo *assembler (NEWBLER v2.0.00.22, Roche/454 Life Sciences) with the default parameters (40 bp overlap; 90% identity), resulting in 82, 134 contigs and 134, 971 singletons (Table 1). We found that some singletons could be aligned to the contigs with 95% percent identity through the entire region except less than 10 bp from both 5' and 3' ends. We used Blast to map 5407 singletons to 2471 NEWBLER contigs. An additional attempt was made to assemble the remaining singletons using MIRA [32]. The recommended parameters for 454 reads were used, resulting in additional 14, 245 contigs and 93, 000 singletons remaining. Of these singletons, 339 reads were mapped to 380 contigs. Therefore, the final number of singletons is 92, 561 (Table 1).

For gene identification, contigs were compared to NCBI-NR protein database, HomoloGene, UniGene (Chicken) [56] databases using BlastX and tBlastX with cut-off e-values of 1e^-5^, 1e^-10^, 1e^-20 ^and 1e^-50^. Databases accessed in January 2010 were used for these analyses. Additionally the contigs were mapped to the draft Anolis lizard genome (AnoCar1.0) [8] and Ensemble annotated gene models using tBLATx and BlastX. Open reading frames (ORFs) were predicted using OrfPredictor [57] with the NR BLAST hits and NCBI's ORF Finder. Databases accessed in August 2010 were used for these analyses.

The NCBI-NR BlastX hits (e-value = 1e^-5^) for the NEWBLER contigs, MIRA contigs, and singletons were used with the program MEGAN [38] to map the sequences to the NCBI taxonomy (databases accessed on January 2010 for BlastX and February 2010 for taxonomy). Sequences that were assigned to the very tips of the branches outside of Chordata were considered to have originated from off-target species (i.e., not from *T. elegans*). MEGAN was used to evaluate the GO annotation (GO Slim) assigned to each term (as of February 2010).

### Additional clustering

We used two methods for additional clustering: homology clustering, and a newly developed method we refer to as graph-clustering or contig-graphs (Figure 1). Homology clustering is the grouping of singletons and contigs based on their BlastX hits in HomoloGene, and the draft lizard gene models (AnoCar1.0). The homology clustering was based on four different cut-off e-values: 1e^-5^, 1e^-10^, 1e^-20 ^and 1e^-50^

The graph-clusters were assembled independent of any comparisons to other databases, but rather solely dependent on the information derived from the original reads. The GS *de novo *(NEWBLER, Roche/454 Life Sciences) assembly program is set up to allow for improved contig alignments when there is abundant alternatively spliced transcripts and gene duplication events by allowing reads to be split into two and each portion assigned to a different contig. We have developed a clustering method based on graph theory that uses this 'historical' information on how the reads were split and assigned into contigs (see Additional file 3 for full description). The underlying algorithm clusters contigs into network graphs where the contigs represent the nodes and the split reads are the edges. These graph-clusters can contain components that represent 1) a single gene with divergent alleles, 2) a single gene with alternatively spliced transcripts, 3) closely related genes within a gene family (gene duplications), or 4) any combination of the three (Additional file 3). In the case of alternatively-spliced transcripts, the nodes indicate exons, and the edges indicate the combinations of how these exons connect in the different transcripts (transcriptional paths). These exons could not be merged further based on similarity to each other. In the case of duplicated genes, parts of the transcripts were highly similar and merged into the same contig, but reads covering the regions of the duplicated genes that have diverged were split into different contigs (nodes). These nodes representing the diverged regions of the duplicated genes were still quite similar (assuming >80% but < 95% sequence identity). This similarity is used to distinguish if a contig-graph represents an alternatively-spliced gene or duplicated genes. Contig-graphs representing divergent alleles from the same gene are distinguished from duplicated genes by assuming that the alleles have > 95% sequence identity (see Additional file 3 for more details on the graph-clustering method).

For each component within a graph-cluster, the BlastX results for its contigs were summarized in order to classify the component into one of five categories. 1) None of the contigs had a homology hit; 2) some of the contigs had a homology hit, but to different files in NCBI; 3) some of the contigs had a homology hit, and they were to the identical file in NCBI; 4) all of the contigs had a homology hit, but to different files in NCBI; 5) all of the contigs had a homology hit, and they were all to the identical file in NCBI. These were summarized for each type of component.

### Sequence variants

Sequence variants (SNPs and INDELs) were identified and their probability of being a 'true' variant based on Bayesian analysis using the program GIGABAYES [58,59]. Since NEWBLER aligns reads allowing gaps instead of substitutions, sequence variant callers cannot identify SNPs and INDELs accurately. MOSAIK [60] was used to realign reads against contigs. Because homopolymer errors are more prevalent with 454 sequences than ABI sequences, this has to be taken into account when calculating probabilities. INDELs called by GIGABAYES were filtered out if they were in homopolymer regions. For high confident sequence variants, we filtered out SNPs and INDELs with read coverage < 5 or > 100 and the probability < 0.9.

The relationship between the number of variants per length of the contig was tested using a regression analysis in R. Contigs in the 99^th ^percentile of number of variants/contig length were considered highly variable. The ratio between transitions and transversions ((TS + 1)/(TV+ 1)) was calculated for each contigs containing SNPs. The one is added to allow the ratio to be calculated for contigs that had a zero for either TS or TV values. For each contig-containing SNP, for which we had a predicted ORF, we calculated the ratio of non-synonymous (Ka) to synonymous (Ks) polymorphisms ((Ka + 1)/(Ks+ 1)). (python script available at http://eco.bcb.iastate.edu/).

### Comparisons between female and male

The sex-of-origin of each read was tracked through the assembly so that the contigs could be classified as being a "female" contig (only containing female reads), a "male" contig (only containing male reads), or a "both" contig (containing both male and female reads) (Figure 1). To identify genes that are expressed by only one of the sexes, we compared the BlastX for each contigs and singleton. For example, if a BlastX hit was found in only female contigs and/or female singletons, and had no homology to male contigs or male singletons at e-values down to 1e^-50^, then it was classified as female-specific. The same process was used to identify male-specific genes. These 190 genes were compared to RefSeq for all vertebrates. Their GO slim assignments for Biological Processes were statistically compared in Blast2Go to test for enrichment of particular GO terms using Fisher's Exact Test.

## Authors' contributions

TSS, SP, and AMB: conceived and designed the research plan. AMB collected animals; TSS and AMB: dissected tissues and isolated the RNA; KM: optimized the normalization and library preparation for Titanium platform and conducted the sequencing; J-HC: designed the bioinformatic analysis strategy and improved the assembly; HT: analyzed homology-based annotation and ORF prediction; YIY: analyzed the graph-clustering and sequence variants; JLV and TSS: conducted Ka/Ks analyses, additional annotation, and metagenomic analysis; TSS: drafted the manuscript. All authors contributed to the interpretation of the results and the content of the final manuscript.

## Supplementary Material

Additional file 1 **Table containing details of the samples used for the sex-specific RNA pools**. Tissue samples of the same type were pooled across individuals (either laboratory or field born animals) for total RNA extraction. Extracted pools of RNA were quantified and the quality checked on the Bioanalyzer. Equal amounts of RNA from each tissue type were pooled by sex. Click here for file

Additional file 2 **Graphs illustrating the size distribution of the reads for each sex**. Length (bp) distribution of reads obtained with the 454 GS-FLX Titanium sequencing. Read number (N) and length (L) in base pairs. A) Female run. B) Male runs. Click here for file

Additional file 3 **Description of NEWBLER assembly and graph-clustering procedure**. Click here for file

Additional file 4 **Pie graphs of GO assignments**. GO slim (level 1, Biological Processes) assignments for all the sequences with annotation, broken down by class of sequences: male singletons, male contigs, both contigs (containing male and female reads), female contigs, female singletons. Click here for file

Additional file 5 **Snake transcripts mapped to coding and non-coding regions of the Anolis lizard draft genome (AnoCar1.0)**. A 2007 Excel file (.xlsx) providing details where the snake transcripts mapped to the Anolis draft genome. See the ReadME tab for description of columns. Click here for file

Additional file 6 **Clustering based on homology and contig-graphs**. A) Distribution of the number of contigs in a HomoloGene accession, and B) the number of HomoloGene accessions that a contig is assigned to, both at e-value = 1e^-20^. C) Distribution of the number of contigs belonging to a graph-cluster. Click here for file

Additional file 7 **Details of variants**. A 2007 Excel file (.xlsx) providing details for the variants (SNPs and INDELs). See the ReadME tab for description of columns. Click here for file

Additional file 8 **Contigs of interest**. A 2007 Excel file (.xlsx) containing the sequences of interest including those that are sex-specific, that have homology to the chicken Z chromosome, those in the 1^st ^percentile of TS/TV ratios, hose in the top 99^th ^percentile of Ka/Ks ratios, and those, in the top 99^th ^percentile of variability (number of variants per bp). See the ReadME tab for description of columns. Click here for file

Additional file 9 **Sex-specific enrichment of GO terms (level 2, Biological Processes) assigned to the 190 sex-specific sequences**. The * indicates the significant over-enrichment of sequences involved in biosynthetic processes in the female-specific sequences (Fisher's Exact Test, FDR <0.006, p-value < 0.0002). Click here for file
