# Supplementary material for: A garter snake transcriptome: pyrosequencing, de novo assembly, and sex-specific differences
Source: BMC Genomics. 2010 Dec 7;11:694. doi: 10.1186/1471-2164-11-694 (PMC3014983; doi:10.1186/1471-2164-11-694)
Supplement: Additional file 1 — Table containing details of the samples used for the sex-specific RNA pools. Tissue samples of the same type were pooled across individuals (either laboratory or field born animals) for total RNA extraction. Extracted pools of RNA were quantified and the quality checked on the Bioanalyzer. Equal amounts of RNA from each tissue type were pooled by sex. [file 1471-2164-11-694-S1.DOC]

### Additional file 1 – Table containing details of the samples used for the sex-specific RNA pools. Tissue samples of the same type were pooled across individuals (either laboratory or field born animals) for total RNA extraction. Extracted pools of RNA were quantified and the quality checked on a Bioanalyzer. Equal amounts of RNA from each tissue type were pooled by sex.

|  | Male Pool | Female Pool |
| --- | --- | --- |
| Tissues(No. Individuals) | blood (14)brain (16)heart (18)kidney (18)liver (18)heart, kidney, liver pool (4)spleen (13)testis (6) | blood (13)brain (15)heart (17)kidney(17)liver (17)heart, kidney, liver pool (4)spleen (12)ovaries (4) |
| Field AnimalsEcotype^1^: Population(No. Individuals) | 14 TotalFL: Christie (3)FL: Gallatin (3)FL: Pikes (3)SL: Mahogany (2)SL: Papoose (3) | 13 TotalFL: Christie (3)FL: Gallatin (2)FL: Pikes (3)SL: Mahogany (3)SL: Papoose (3) |
| Laboratory AnimalsEcotype^1^: Ancestral Population(No. Individuals and Treatment^2^) | 4 TotalFL: Merrill (1 cool, 1 warm)SL: Papoose (1 cool, 1 warm) | 4 TotalFL: Merrill (1 cool, 1 warm)FL: ELFS (1 cool)SL: Mahogany (1 warm) |
| Total No. Populations Represented | 6 | 7 |
| Total No. Individuals Represented | 18 | 17 |
| Pool Bioanalyzer RIN | 8.4 | 9.1 |

^1^ FL = fast-living ecotype, SL= slow-living ecotype as described in text.

^2^Thermal experiment with cool and warm temperature treatments.
