# Supplementary material for: A garter snake transcriptome: pyrosequencing, de novo assembly, and sex-specific differences
Source: BMC Genomics. 2010 Dec 7;11:694. doi: 10.1186/1471-2164-11-694 (PMC3014983; doi:10.1186/1471-2164-11-694)
Supplement: Additional file 2 — Graphs illustrating the size distribution of the reads for each sex. Length (bp) distribution of reads obtained with the 454 GS-FLX Titanium sequencing. Read number (N) and length (L) in base pairs. A) Female run. B) Male runs. [file 1471-2164-11-694-S2.DOC]

Additional file 2 – Distribution of reads.

Length (bp) distribution of reads using the 454 GS-FLX Titanium Chemistries for sequencing. Read number (N) and length (L) in base pairs. A) Female run. B) Male runs.


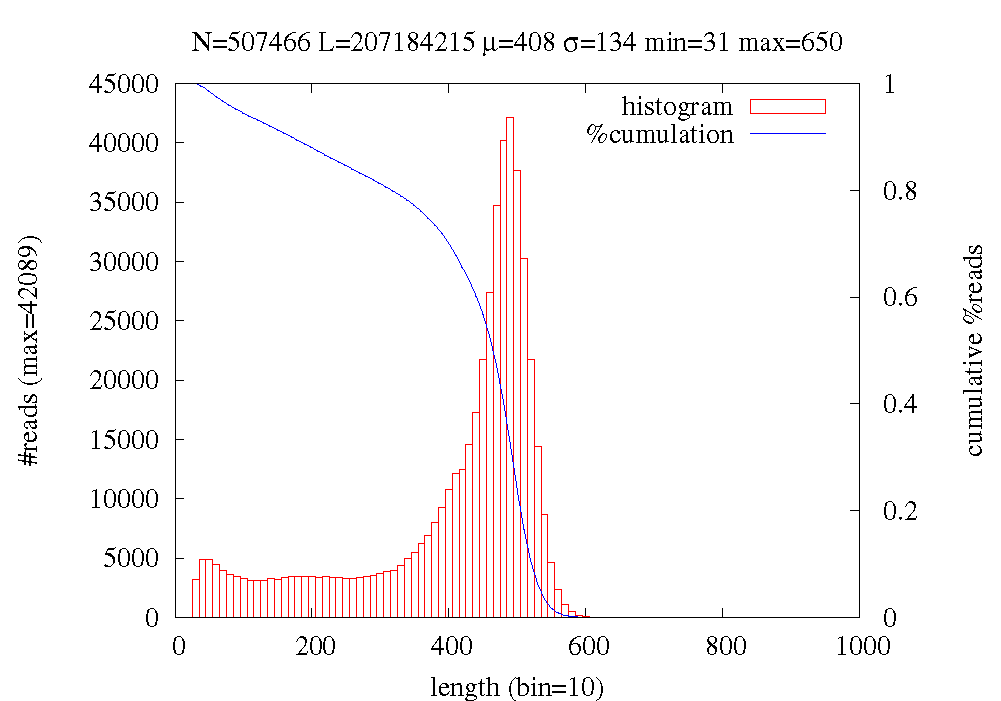
A

**B**

**
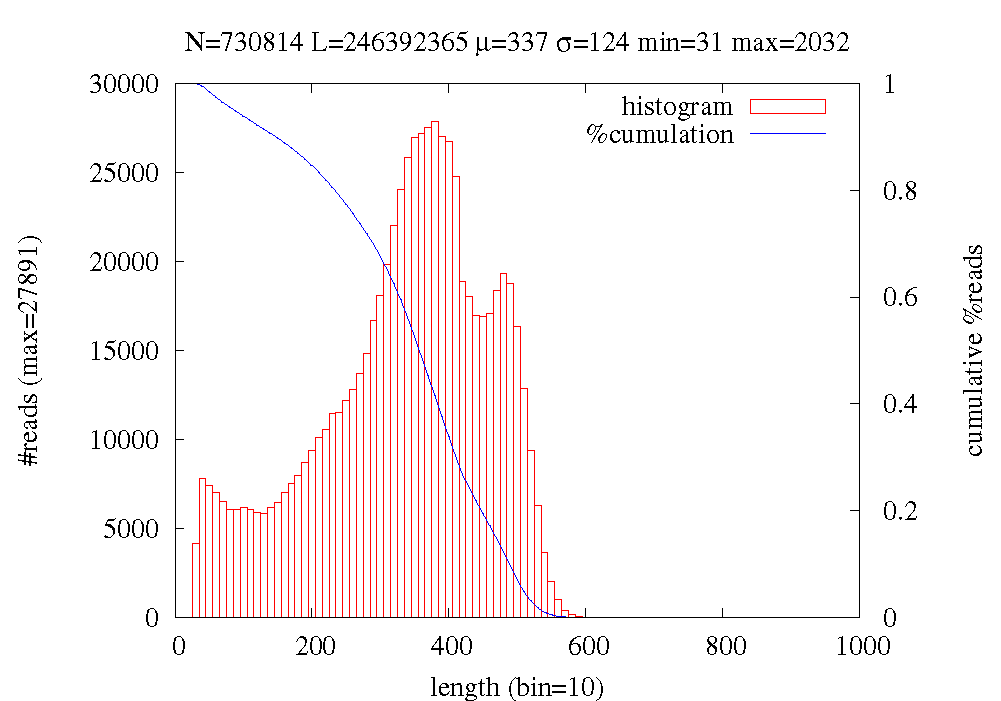
**
