# Supplementary material for: A garter snake transcriptome: pyrosequencing, de novo assembly, and sex-specific differences
Source: BMC Genomics. 2010 Dec 7;11:694. doi: 10.1186/1471-2164-11-694 (PMC3014983; doi:10.1186/1471-2164-11-694)
Supplement: Additional file 3 — Description of NEWBLER assembly and graph-clustering procedure. [file 1471-2164-11-694-S3.PDF]

CONTIG CLUSTERING USING 454 SPLIT READS

JEONG-HYEON CHOI

The Center for Genomics and Bioinformatics, Indiana University, Bloomington, IN 47405

Newbler, the 454 GS Assembler, splits sequence reads at the boundaries of repeats and alternative splice sites. In other words, *split reads* are defined as reads of which parts are placed in different contigs. In transcriptome assemblies, we have observed 3 cases: alternative splicing transcripts, duplicated genes and sequence variants.

1. Alternative Splicing

In Figure 1, a gene has two alternative splicing transcripts as represented by the different colored lines. Since transcripts, not genes, are amplified for sequencing samples, sequence reads come from transcripts as represented by the lines in Figure 1. Reads within each exon are the same except for sequencing errors and are assembled into the same contig by Newbler. Let c_1, c_2 and c_3 be contigs for the exons a, b and c respectively as shown in Figure 2. These contigs cannot be further merged because the reads r_1, r_2 and r_3 cause different merge paths. The split reads can be used to build

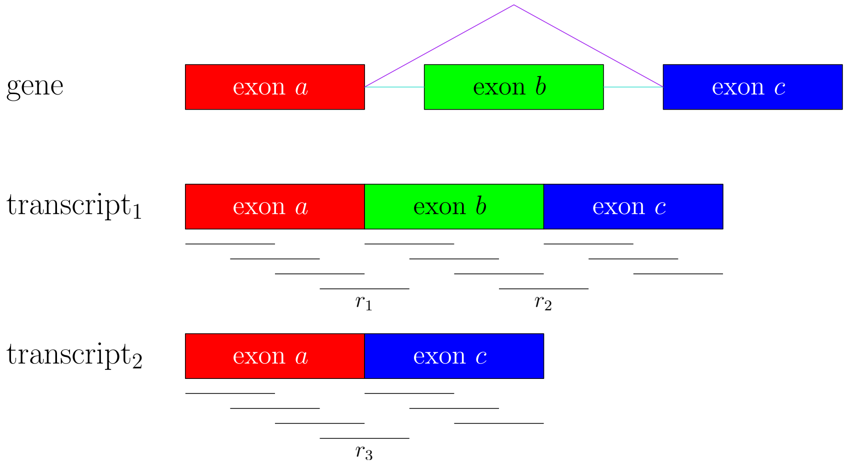

Figure 1. Gene with two alternative splicing transcripts. The lines represent reads of which r_1, r_2 and r_3 are sequenced from the boundaries of two exons.

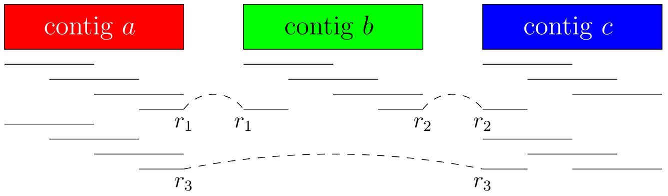

Figure 2. Assembly made by Newbler where each contig represents an exon and the reads r_1, r_2 and r_3 are split reads.

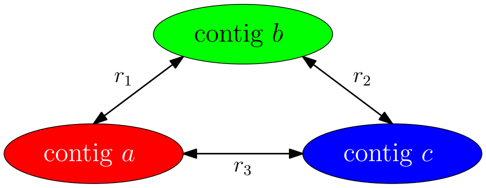

FIGURE 3. Contig graph where each node represents a contig and an edge represents a split read.

a contig graph. In Figure 3, the contigs a, b and c are connected to each other by the reads r_1, r_2 and r_3. From the figure, we can identify two differently transcriptional paths (transcripts) from the contig a to the contig c.

2. Duplicated Genes

Recently duplicated genes could share highly similar regions ($\geq$ 95% identity) with each other, which could be assembled into the same contigs. Figure 4 shows the exons a and c are almost identical and assembled into the contigs a and c respectively as shown in Figure 5. Since the exons b_1 and b_2 are diverse, they are assembled into different contigs b_1 and b_2. Similar to an alternative splicing gene, the split reads r_1, r_2, r_3 and r_4 are used as edges in a contig graph (Figure 6). Therefore, there are two paths in the graph from the contig a to the contig c, but this case is different from alternative splicing genes. If the contigs b_1 and b_2 are similar enough to be aligned, say $\geq$ 80%, this graph can be judged to have duplicated genes. Otherwise, without the genome structure, we do not know if the graph results from alternative splicing or duplicated genes.

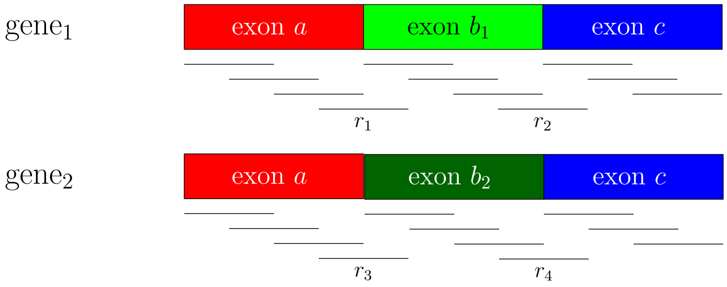

FIGURE 4. Duplicated genes with highly similar regions (exons a and b) and a diverse region (exons b_1 and b_2). The reads r_1, r_2, r_3 and r_4 are sequenced from the boundaries of two exons.

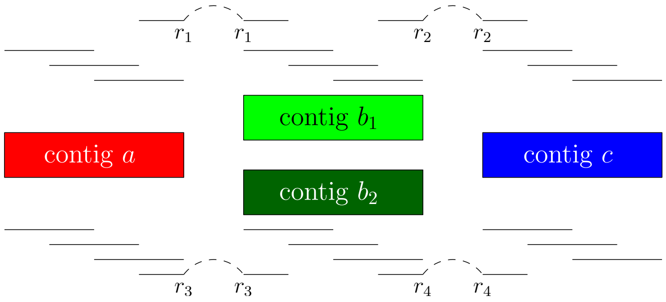

FIGURE 5. Assembly made by Newbler where each contig represents an exon and the reads r_1, r_2, r_3 and r_4 are split reads.

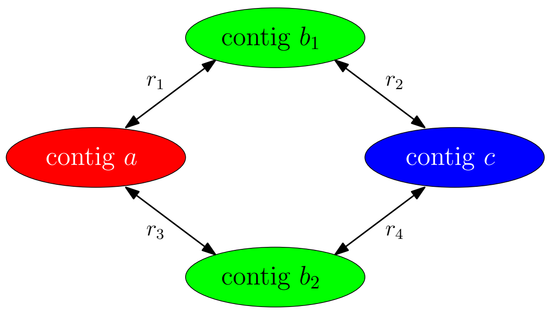

FIGURE 6. Contig graph where each node represents a contig and an edge represents a split read.

3. DIFFERENT ALLELES

If a pooled sample from different alleles and tissues is sequenced together, a gene could have sequence variants such as SNPs and indels among sequence reads. Although they are almost identical except for a few bases, assemblers could separate them to different contigs because modern assemblers have a mechanism to resolve collapses due to repeats. In Figure 7, a gene has an exon with 2 SNPs, A–T and C–G. If assemblers such as Newbler assume that the sequenced sample has a single allele and the exon b is a repeat, then the exon is assembled into different contigs like the duplicated genes in Figure 5. Finally the reads r_1, r_2, r_3 and r_4 make a contig graph as shown in Figure 6. However, the contigs b_1 and b_2 are almost identical except for the 2 SNPs. Contigs separated by different alleles are distinguished from duplicated genes by measuring the percent identity of the contigs b_1 and b_2. If the percent identity is greater than a cutoff, say 95%, then the contig graph results from different alleles. Otherwise, it is regarded as duplicated genes. It is worthwhile to note that the graph is more likely to be an alternative splicing gene if the contigs have no similarity.

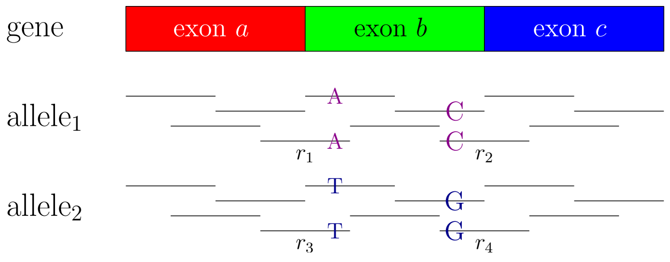

FIGURE 7. Gene having an exon b with 2 SNPs.
