# Supplementary material for: A garter snake transcriptome: pyrosequencing, de novo assembly, and sex-specific differences
Source: BMC Genomics. 2010 Dec 7;11:694. doi: 10.1186/1471-2164-11-694 (PMC3014983; doi:10.1186/1471-2164-11-694)
Supplement: Additional file 4 — Pie graphs of GO assignments. GO slim (level 1, Biological Processes) assignments for all the sequences with annotation, broken down by class of sequences: male singletons, male contigs, both contigs (containing male and female reads), female contigs, female singletons. [file 1471-2164-11-694-S4.DOC]

**Additional file 4– Pie graphs of GO assignments.**

GO slim (level 1, Biological Processes) assignments for all the sequences with annotation, broken down by class of sequences: male singletons, male contigs, both contigs (containing male and female reads), female contigs, female singletons.

**Female**

**Singletons**

**Female**

**Contigs**

**Both Contigs**

**Male**

**Contigs**

**Male**

**Singletons**

**All Sequences**

| **metabolic process** | | **regulation of biological process** | | | | **transport** | | **cellular component organization** | | | | **multicellular organismal development** | | |
| --- | --- | --- | --- | --- | --- | --- | --- | --- | --- | --- | --- | --- | --- | --- |
| **cellular homeostasis** | **response to stress** | | **cell cycle** | **cell differentiation** | | | **reproduction** | | **cell communication** | | **anatomical structure morphogenesis** | | | |
| **response to biotic stimulus** | | **response to external stimulus** | | | **cell proliferation** | | | **symbiosis** | | **behavior** | | | **cell recognition** | **growth** |
