# Supplementary material for: A garter snake transcriptome: pyrosequencing, de novo assembly, and sex-specific differences
Source: BMC Genomics. 2010 Dec 7;11:694. doi: 10.1186/1471-2164-11-694 (PMC3014983; doi:10.1186/1471-2164-11-694)
Supplement: Additional file 6 — Clustering based on homology and contig-graphs. A) Distribution of the number of contigs in a HomoloGene accession, and B) the number of HomoloGene accessions that a contig is assigned to, both at e-value = 1e-20. C) Distribution of the number of contigs belonging to a graph-cluster. [file 1471-2164-11-694-S6.DOC]

**Additional file 6 - Clustering based on homology and contig graphs.**

A) Distribution of the number of contigs in a HomoloGene accession, and B) the number of HomoloGene accessions that a contig is assigned to, both at e-value =1e-20.

C) Distribution of the number of contigs belonging to a graph-cluster.

A

B

C
