# Supplementary material for: A garter snake transcriptome: pyrosequencing, de novo assembly, and sex-specific differences
Source: BMC Genomics. 2010 Dec 7;11:694. doi: 10.1186/1471-2164-11-694 (PMC3014983; doi:10.1186/1471-2164-11-694)
Supplement: Additional file 9 — Sex-specific enrichment of GO terms (level 2, Biological Processes) assigned to the 190 sex-specific sequences. The * indicates the significant over-enrichment of sequences involved in biosynthetic processes in the female-specific sequences (Fisher's Exact Test, FDR <0.006, p-value < 0.0002). [file 1471-2164-11-694-S9.DOC]

Additional file 9 – Sex-specific enrichment of GO terms (level 2, Biological Processes) assigned to the 190 sex-specific sequences. The * indicates the significant over-enrichment of sequences involved in biosynthetic processes in the female-specific sequences (Fisher’s Exact Test, FDR <0.006, p-value < 0.0002).

*
